# Validation of the AlamarBlue® Assay as a Fast Screening Method to Determine the Antimicrobial Activity of Botanical Extracts

**DOI:** 10.1371/journal.pone.0169090

**Published:** 2016-12-29

**Authors:** Olaf Tyc, Laura Tomás-Menor, Paolina Garbeva, Enrique Barrajón-Catalán, Vicente Micol

**Affiliations:** 1 Netherlands Institute of Ecology (NIOO-KNAW), Department of Microbial Ecology, Wageningen, The Netherlands; 2 Instituto de Biología Molecular y Celular (IBMC), Universidad Miguel Hernández (UMH), Avenida de la Universidad s/n, E-03202 Elche, Alicante, Spain; 3 INVITROTECNIA S.L., Santiago Grisolía 2, Tres Cantos, Madrid, Spain; National University of Ireland—Galway, IRELAND

## Abstract

Plant compounds are a potential source of new antimicrobial molecules against a variety of infections. Plant extracts suppose complex phytochemical libraries that may be used for the first stages of the screening process for antimicrobials. However, their large variability and complexity require fast and inexpensive methods that allow a rapid and adequate screening for antimicrobial activity against a variety of bacteria and fungi. In this study, a multi-well plate assay using the AlamarBlue® fluorescent dye was applied to screen for antimicrobial activity of several botanical extracts and the data were correlated with microbial colony forming units (CFU). This correlation was performed for three pathogenic model microorganisms: *Escherichia coli* (Gram negative bacteria), *Staphylococcus aureus* (Gram positive bacteria) and for the yeast-like fungi *Candida albicans*. A total of ten plant extracts from different Mediterranean plants, including several *Cistus* and *Hibiscus* species, were successfully tested. HPLC-DAD-ESI-MS/MS analysis was utilized for the characterization of the extracts in order to establish structure-activity correlations. The results show that extracts enriched in ellagitannins and flavonols are promising antibacterial agents against both Gram positive and Gram negative bacteria. In contrast, phenolic acids, anthocyanidins and flavonols may be related to the observed antifungal activity.

## Introduction

Despite the numerous advances made in medicine and pharmacology, there is still a need for new antimicrobial agents, especially due to outbreak of multi-drug-resistant forms of both Gram negative and Gram positive bacteria to front-line antibiotics [1–4]. Antibiotic resistance has become one of the major sanitary problems worldwide and disseminate rapidly amongst patients in healthcare facilities. Although resistant bacteraemia rates seemed to decrease in several EU countries within the past years, the European Medicine Agency (EMA) estimates that these infections provoke a sanitary additional cost around 1.5 billion euro in the EU [5]. Only two families of new antibiotics (lipopeptides and oxazolidinones) have reached clinic practice in the last 40 years [5]. Given that fact, the actual therapeutic arsenal is clearly insufficient and there is an urgent need for new treatments for these infections, in particular agents that suppress or abrogate the emergence of drug resistance.

Plant compounds are a potential source of new antimicrobial molecules against a range of different pathogens. Nevertheless, the complexity of plant metabolites and the difficulty to isolate pure compounds makes it challenging to extrapolate the results of the antimicrobial screening studies. Currently, several plant polyphenolic compounds have demonstrated broad spectrum antimicrobial biological activity [6–8]. In this work, four species of *Cistus* genus (*Cistus ladanifer*, *Cistus clusii*, *Cistus albidus* and *Cistus salviifolius*) and four other plant species (*Hypoxis rooperi*, *Hibiscus sabdariffa*, *Hibiscus arnottianus* and *Lippia citriodora)* have been selected for antimicrobial studies based on previous studies performed in our lab and ethnobotanical knowledge.

The antimicrobial activity of extracts derived from *Cistaceae* against both Gram positive and Gram negative bacteria has been reported [9–11]. Among them, *Cistus salviifolius* aqueous extract, which mostly contains ellagitannins and flavonoids, was reported to be particularly potent [11, 12]. The strong antimicrobial activity of the polyphenolic extract from *Cistus salviifolius* against *S*. *aureus* has been related with the synergic pharmacological interaction between ellagitannins and flavonols [13]. The antimicrobial activity of *Hypoxis rooperi* extract have also been related to its polyphenolic content [14]. On the other hand, the antimicrobial activity of *Lippia citriodora* has also been studied especially against Gram positive bacteria [15–17]. However, data about antimicrobial activity of white roselle (*Hibiscus arnottianus)* has not been published so far.

The microplate AlamarBlue assay (MABA) is a sensitive, rapid, inexpensive, and nonradiometric method, which evaluates metabolic function and cellular health and offers the potential for screening large numbers of antimicrobial compounds. MABA has offered several advantages compared to other radiometric methods for high-throughput screening [18] or for MIC50 determination [19]. MABA major advantage is that growth can be evaluated fluorometrically or spectrophotometrically or visually [19], the last without the use of specialized equipment.

The aim of the present study was to apply MABA to screen for antimicrobial activity of ten different plant extracts and to correlate it with colony forming units (CFU/mL) measurement. In addition, the composition of the main polyphenolic constituents of the plant extracts was determined by using high performance liquid chromatography coupled to electrospray ionization and ion trap tandem mass spectrometry (HPLC-ESI-IT-MS/MS) in order to identify the main compounds of those extracts showing the highest activity against three different microorganisms. The study was performed using a total of ten plant extracts and three clinically relevant strains, Gram negative (*Escherichia coli*) and Gram-positive bacteria (*Staphylococcus aureus*) and the diploid fungus *Candida albicans*. With this method kinetics information about the inhibitory effects of the different extracts on the microbial growth was obtained.

## Material and Methods

### Plant collection, extraction and fractionation

The *Cistus* species were selected based on our previous expertise on these plants [11]. *Cistus* plant material was obtained from natural reservations of Ciudad Real (*Cistus*. *ladanifer*; Puerto Llano, 38°41'18.6"N 4°01'26.9"W), Valencia (*Cistus clusii*, Parque Natural Sierra Calderona, 39°41'31.5"N 0°28'52.7"W) and Alicante (*Cistus salviifolius*, Monóvar, 38°25'22.9"N 0°49'57.1"W) provinces (Spain) in early spring, according to flowering time and reserves’ rules and were extracted as previously reported [9, 11, 12]. Briefly, *Cistus* plants were extracted with distilled water at a temperature below 65°C, with gentle agitation for approximately 2 h and using a plant–solvent ratio of 1:5. In the case of *C*. *ladanifer*, the extract was obtained by direct aqueous extraction and latter affinity chromatography was performed as previously described [11]. *Hibiscus arnottianus* specimens were collected from the coast of Granada (37°06'19.0"N 3°33'44.0"W) (Spain) and extracted as reported [20]. Permissions were obtained from local authorities (Conselleria de Agricultura de la Generalitat Valenciana and Consejería de Agricultura de la Comunidad Autónoma de Castilla La Mancha). *Cistus* species are not in danger and are not protected according to Spanish legislation. All the other plant materials were obtained from commercial source. All the plant materials were properly identified and labeled by the authors and qualified personnel and deposited in the Universidad Miguel Hernández facilities (refs. CS30082013, CC30082013, CL30082013, CA30082013 and HA30082013). *Lippia citriodora* extracts PLX10 and PLX32 (containing 10 and 32% verbascoside w/w respectively) and *Hypooxis rooperi* extract (containing 11% w/w polyphenols as measured by Folin-Ciocalteu assay) were kindly provided by Monteloeder S.L. *Hibiscus sabdariffa* extract (containing 6% w/w polyphenols by Folin-Ciocalteu) was provided by Nutrafur S.L. The polyphenolic enriched extract of *H*. *sabdariffa* was obtained as described [20].

### HPLC–DAD–MS/MS

The composition of the extracts that were not previously characterized was analyzed by HPLC-DAD-ESI-MS/MS technique with an Agilent LC 1100 series (Agilent Technologies, Inc., Palo Alto, CA, USA) controlled by the Chemstation software and equipped with a pump, autosampler, column oven and UV–vis diode array detector. The HPLC instrument was coupled to an Esquire 3000+ (Bruker Daltonics, GmbH, Germany) mass spectrometer equipped with an ESI source and ion-trap mass analyzer, and operated by Esquire control and data analysis software. A Phenomenex Kinetex 2.6 μm C18, 150×4.6 mm column was used for the separation. Detector conditions for DAD and ESI were applied as previously described [12]. The mobile phases consisted of water:acetonitrile (90:10, v/v) with 1% of formic acid (A) and acetonitrile (B). Gradient started with 5% B, 30%B at 20 min, 90% B at 30 min, 5% B at 35 min and 5 more minutes for re-equilibration. The flow was set to 0.5 mL/min. Identification of the compounds was made by HPLC-DAD analysis, using Data Analysis Software v3.4 (Bruker Daltonics) software and comparing the retention time, UV spectra and MS/MS data of the peaks in the samples with those of authentic standards or data reported in the literature.

### Total polyphenolic content

The total polyphenolic content was determined using the Folin–Ciocalteu method [21] with gallic acid (SIGMA–ALDRICH, Europe) as a standard. The absorbance measurements were performed using a UV–VIS spectrophotometer (Cecil 2041 2000 Series, UK).

### Preculturing of target organisms

Two bacterial target organisms were applied in MABA: (1) *Escherichia coli* WA321 (DSMZ # 4509) as Gram negative model bacteria and (2) *Staphylococcus aureus* 533R4 Serovar 3 (DSMZ # 20 231) as Gram positive model bacteria. To test for antimicrobial activity against yeast-like microorganisms, the fungi *Candida albicans* BSMY 212 (DSMZ # 10697) was utilized. The two bacterial strains *E*. *coli* WA321 and *S*. *aureus* 533R4 were precultured from -80°C glycerol stocks on lysogeny broth according to Bertani (LB) agar plates (10.0 g/L Merck NaCl, 10 g/L BACTO™ Tryptone, 5 g/L BACTO™ Yeast extract, 20 g/L Merck Agar) [22] and incubated at 37°C for 24 h prior application. *C*. *albicans* was precultured from -80°C glycerol stocks on YEPD plates (20.0 g/L Merck Dextrose, 20.0 g/L BACTO™ Peptone, 10.0 g/L BACTO™ Yeast extract, 20 g/L Merck Agar) [22] and incubated at 37°C for one day prior application. All target microorganisms used in this study are summarized in Table 1.

**Table 1:** Information about the used strains.

Species	Strain number	Assay function
*Candida albicans* BSMY 212	DSMZ 10697	Human pathogen model organism for yeast-like fungi
*Escherichia coli* WA321	DSMZ 4509	Human pathogen model organism for Gram negative bacteria
*Staphylococcus aureus* 533R4 Serovar 3	DSMZ 20231	Human pathogen model organism for Grampositive bacteria

Target strains utilized in MABA for the detection of antimicrobial activity of the botanical extracts.

### Microplate AlamarBlue^®^ assay (MABA)

**Fig 1:**
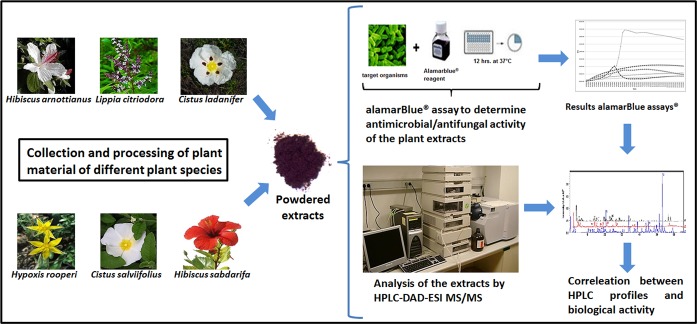
Workflow of the antimicrobial screening. From left to right, different plant species were collected from the natural reservation of Ciudad Real, Valencia and Alicante provinces (Spain), plant material was processed and powdered extracts were made. Extracts were used in MABAs at different concentrations to determine the antimicrobial/antifungal activity. To establish the correlation between the antimicrobial activity of the extracts and their composition using HPLC-DAD-ESI-MS/MS analysis was performed.

For the determination of antimicrobial activity of each plant extract, MABAs were performed [19, 23, 24]. Stock solutions of all extracts at a concentration of 20 mg/mL were freshly prepared on the same day of the S1MABA by re-suspending 200 mg powder of each plant extract in 10 mL of sterile MQ- water. Solutions were vigorously shaken for 10 min in a vertical shaker at 350 rpm followed by centrifugation for 10 min at 1,000 x g. After centrifugation the supernatant was filter sterilized through a 0.2 μm filter (GE™ Whatman™; Puradisc FP 30 cat# 10 462 200). The sterilized stock solutions were diluted into 5 mL liquid LB or YEPD media to a final working concentration of 500 μg/mL, 1 mg/mL or 2 mg/mL. The overview of the workflow is shown in Fig 1.

The growth rates were monitored in the presence of the different extracts and compared to the growth of the control (the tested microorganism in the absence of antimicrobial extract). The following antibacterial agents were applied in the positive assay controls: kanamycin 50 μg/mL, tetracycline 15 μg/mL, (Sigma-Aldrich product # K-4000, T-7660), ampicillin 50 μg/mL (GIBCO-Life Technologies cat# 11593–027). Delvocid® (DSM Food Specialties, The Netherlands) was used as antifungal agent. The AlamarBlue® dyeing solution (life technologies™, USA cat# DAL1025) was aliquoted prior every assay and stored at 4°C. For the measurement of relative fluorescence units (RFU), black 96-well plates (Nunc Microwell™, Polysorp™ F96 cat# 437112) were prepared by dispensing 200 μl of each treatment mixture containing the extract (at concentrations of either 500 μg/mL, 1 mg/mL or 2 mg/mL, selected on our knowledge on antimicrobial extracts), AlamarBlue® 10% (v/v) and one of the target microorganisms (*E*. *coli* WA321, *S*. *aureus* 533R4 or *C*. *albicans* BSMY212). The final density of microorganisms was set to an OD_600_ of 0.002 corresponding to 6.4 x 10^5^ CFU/mL (*E*. *coli* WA321), 4.0 x 10^5^ CFU/mL (*S*. *aureus* 533R4) or 1.6 x 10^4^ Cells/mL (*C*. *albicans* BSMY 212). All extract treatments were measured in 10 replicates and positive growth controls were measured in 10 replicates. The positive assay controls (microorganism plus antibiotics) were measured in triplicates. To normalize the background fluorescence the culture media plus plant extract solution were measured in duplicates. After each sample was added to the 96 well-plate, the fluorescence measurement was started on a BioTek Synergy™ HT Multi-Mode Microplate Reader (Beun de Ronde Life Sciences, The Netherlands) with the following settings: 12 hours of measurement with an interval of 15 minutes, excitation / emission wavelengths of 530 nm and 590 nm respectively. Measurement sensitivity was set to 40 and delay between each sample measurement was set to 50 ms, shaking intensity was set to one and the duration to 300 s before each measurement. The entire assay was performed at 37°C for 12 h. For greater clarity in the figures errors were not represented. Standard deviation values, calculated using Microsoft Excel 2016, were always below 13.9% for *S*. *aureus*, 12.2% for *E*. *coli* and 5.5 for *C*. *albicans* experiments. A comparison of the different antimicrobial compounds used as a control is shown in S2 Fig. Only kanamycin was shown in the figure of the antimicrobial capacity of the extracts for the sake of clarity.

### Estimation of the correlation RFU/CFU

To estimate the correlation between Relative Fluorescence Units (RFU) and Colony Forming Units (CFU) of each target microorganism, MABAs were performed in black 96-well plates (Nunc Microwell™, Polysorp™ F96 cat# 437112) by inoculating the target microorganisms at the same concentration as in the antimicrobial screening assays and by using the identical procedure. MABAs were performed for 6 hours and samples of 100 μL for viable plate counting were taken every 2 hours (T1 = 2h, T2 = 4h, T3 = 6h). Samples for viable counting were plated in triplicates either on LB-A or YEPD media plates and incubated at 37°C overnight. On the next day the viable colonies were counted and the correlation between relative fluorescence units (RFU) and colony forming units (CFU) of each microorganism was estimated. The correlation quotient (R^2^ value) between RFU and CFU was calculated with Microsoft Excel® and is shown in S1 Fig for each strain.

## Results

### Validation of MABA for bacterial growth monitoring

The first step was to validate MABA as an adequate screening method for testing antimicrobial activity based on bacterial growth monitoring. For this, a correlation between MABA results (expressed in relative fluorescent units, RFU) and the colony forming units (CFU) was performed. A good correlation was obtained for all tested microbial strains with the following R^2^ values: 0.9823 for *C*. *albicans* BSMY 212, 0.9966 for *S*. *aureus* 533R4 and 0.9151 for *E*. *coli* WA 321 (S1 Fig). Once the method was validated, antimicrobial tests were performed to screen for extracts with the highest activity among the ten extracts utilized in this study (Table 2). All extracts were tested at concentration of 2 mg/mL against *E*. *coli*, *S*. *aureus* and *C*. *albicans*. Six extracts revealing good activity were selected and tested again at lower extract concentration (1 mg/mL). Finally those extracts that exhibited the strongest antimicrobial activity were selected and tested at a lower concentration, ~ 500 μg/mL. Table 3 summarizes the plant extracts selected for each screening steps.

**Table 2:** Plant extracts used in the antimicrobial study.

Extract	Abbreviation	References	Main compounds	Total polyphenols^#^*
***Hibiscus sabdariffa***	HS	[20, 25]	Hibiscus acid, chlorogenic acid	5.42 ± 0.15
**Polyphenolic enriched *H*. *sabdariffa***	HSP	[20]	Delphinidin-3-sambubioside, cyanidin-3-sambubioside, chlorogenic acid, quercetin-3-sambubioside	33.88 ± 0.88
***Hibiscus arnottatianus***	HA	—*	Chlorogenic acid, quercetin-3-rutinoside	2.10 ± 0.07
***Lippia citriodora* (10% verbascoside)**	PLX10	[26, 27]	Verbascoside	6.37 ± 0.35
***Lippia citriodora* (32% verbascoside)**	PLX32	[26, 27]	Verbascoside	37.86 ± 0.40
***Cistus albidus***	CA	[11, 12]	Catechin, quercetin, kaempferol	19.72 ± 0.74
**Polyphenolic enriched *Cistus ladanifer***	CL	—*	Ellagitannins, flavonols	30.84 ± 0.92
***Cistus clusii***	CC	[11, 12]	Apigenin, kaempferol	15.47 ± 0.45
***Cistus salviifolius***	CS	[11, 12]	Ellagitannins, flavonols and phenolic acids	45.13 ±0.32
***Hypooxis rooperi***	HR	[10]	Hypoxoside	10.11 ± 0.88

The references in which the composition of the extracts was previously described are included.

* Extracts without references are characterized in the present study.

^#^ Total polyphenols ± standard deviation (SD) is expressed in g of gallic acid equivalents/100 g dry weight.

**Table 3:** Selection of extracts.

	2 mg/mL	1 mg/mL	0.5 mg/mL
***S*. *aureus***	All extracts	CS, PLX32,CL, HR, HA, HSP	CS, CL, HR, HSP
***E*. *coli***	All extracts	HS, CS, CL, HR, HA, HSP	CS, CL, HSP
***C*. *albicans***	All extracts	HS, PLX32, CL, HR, HA, HSP	HS, HA, HSP

Botanical extracts selected in each one of the screening steps and the concentration used.

#### *S*. *aureus* microbial growth inhibition

**Fig 2:**
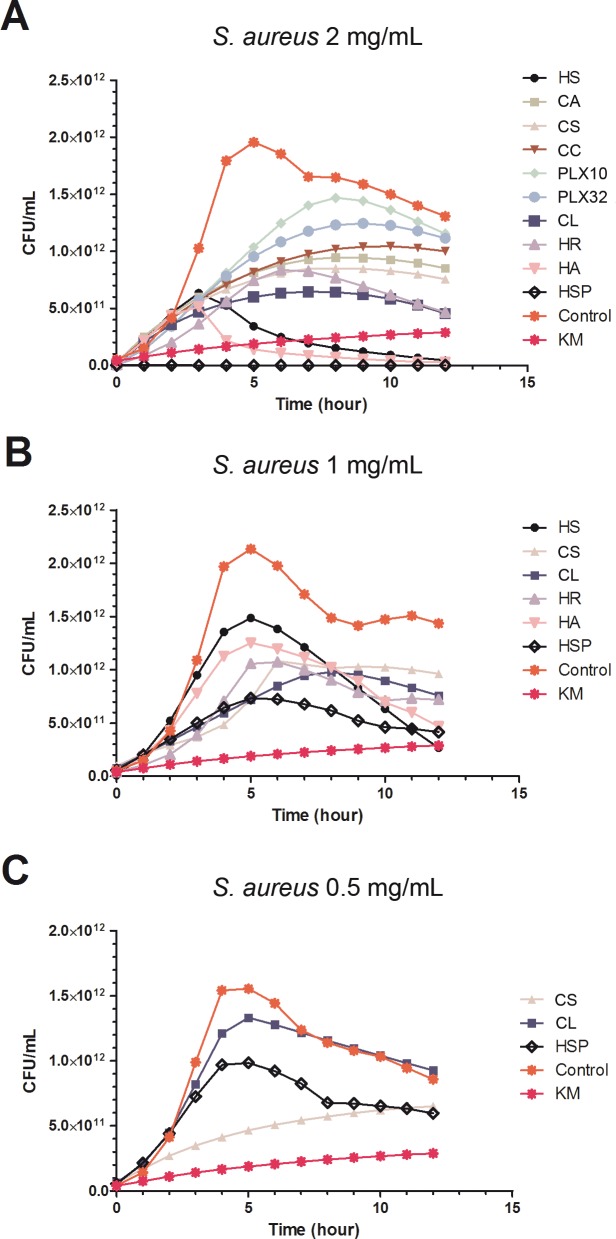
**Plots for MABA experiments against *S*. *aureus* Gram positive bacteria using different concentration of the extracts**: 2 mg/mL (A), 1 mg/mL (B) and 0.5 mg/mL (C). Points represent the mean of 12 replicates. Standard error was calculated for each point (data not shown). KM, kanamycin.

The plant extracts were tested against *S*. *aureus* at a concentration of 2 mg/mL and all extracts exhibited antimicrobial activity at this concentration compared to the control (Fig 2A). Kinetics and endpoint values differed between most of the extracts (Fig 2A). The extracts HS, CS, CL, HR, HA and HSP revealed the highest level of inhibition and were selected for testing at a lower concentration (1 mg/mL). In this second test, although all extracts were still active, strong kinetic differences between extracts was observed (Fig 2B). Despite the HS, HA and HR extracts showed consistent and important growth rate at 5 hours of incubation, i.e. within the bacterial growth log phase, very low endpoint values were observed after 12 hours of incubation, indicating that longer times were required to observe inhibition of microbial growth. These extracts were not selected since their growth inhibitory activity was only shown at nutrient depletion phase. Consequently, we only selected extracts CS, CL and HSP for the last screening step against *S*. *aureus* (Fig 2C) due to their potent effect on the microbial growth at the log phase. CL extract seemed to lose its antimicrobial activity at this concentration, however, HSP and CS retained activity. Only CS extract showed a significant growth inhibitory activity within the exponential growth phase of *S*. *aureus*. For the HSP extract more than 6 hours were needed to inhibit bacterial growth.

#### *E*. *coli* microbial growth inhibition

**Fig 3:**
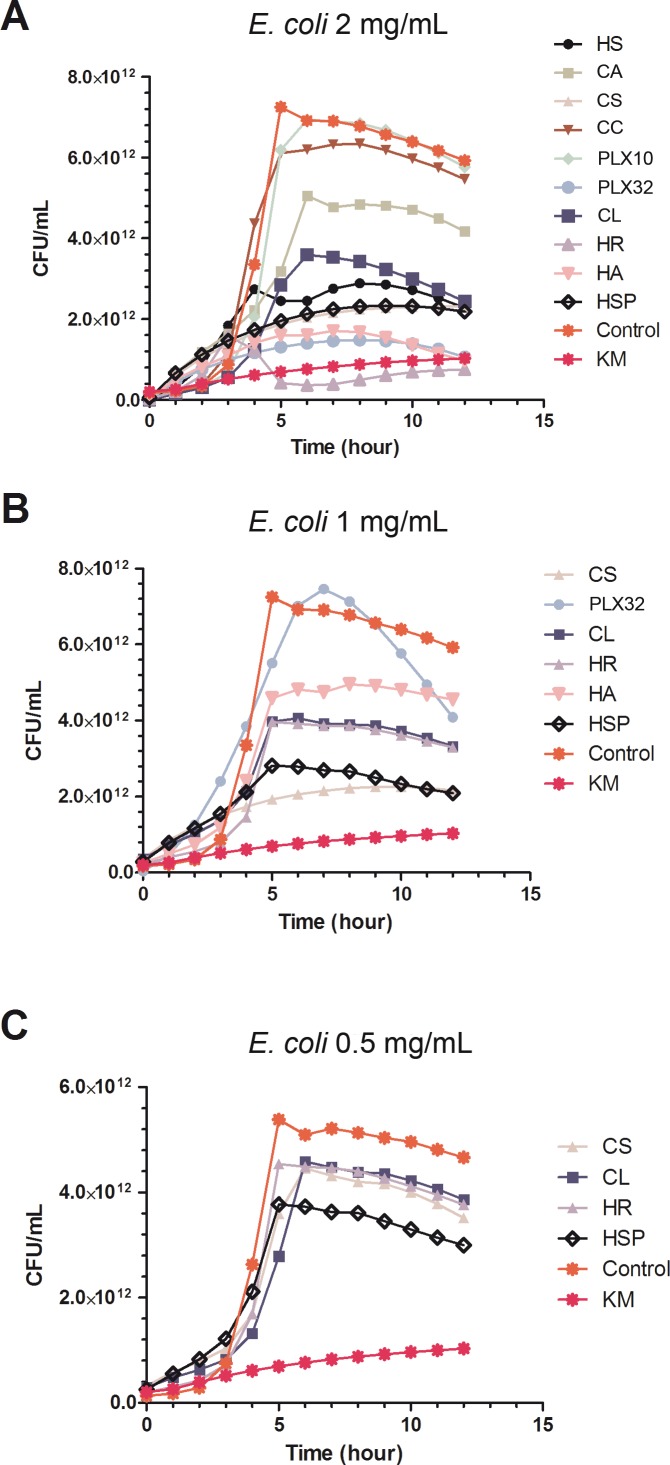
**Plots for MABA experiments against *E*.*coli* Gram negative bacteria using different concentration of the extracts**: 2 mg/mL (A), 1 mg/mL (B) and 0.5 mg/mL (C). Points represent the mean of 12 replicates. Standard error was calculated for each point (data not shown). KM, kanamycin.

Fig 3A shows the growth inhibition of *E*. *coli* by all the extracts tested at concentration of 2 mg/mL. The extracts CA, CC and PLX10 revealed very low activity and were discarded for further tests. The other six extracts were tested again at a concentration of 1 mg/mL (Fig 3B). PLX32 extract lose its antimicrobial activity at this concentration whereas HR and CL retained some of its growth inhibitory activity. On the contrary, CS and HSP showed strong inhibitory activity even within the log phase. Therefore these four extracts were selected for the next step. At 0.5 mg/mL, the four selected extracts showed similar kinetic behavior but only HSP exhibited significant inhibitory activity compared to the control (Fig 3C).

#### *C*. *albicans* microbial growth inhibition

**Fig 4:**
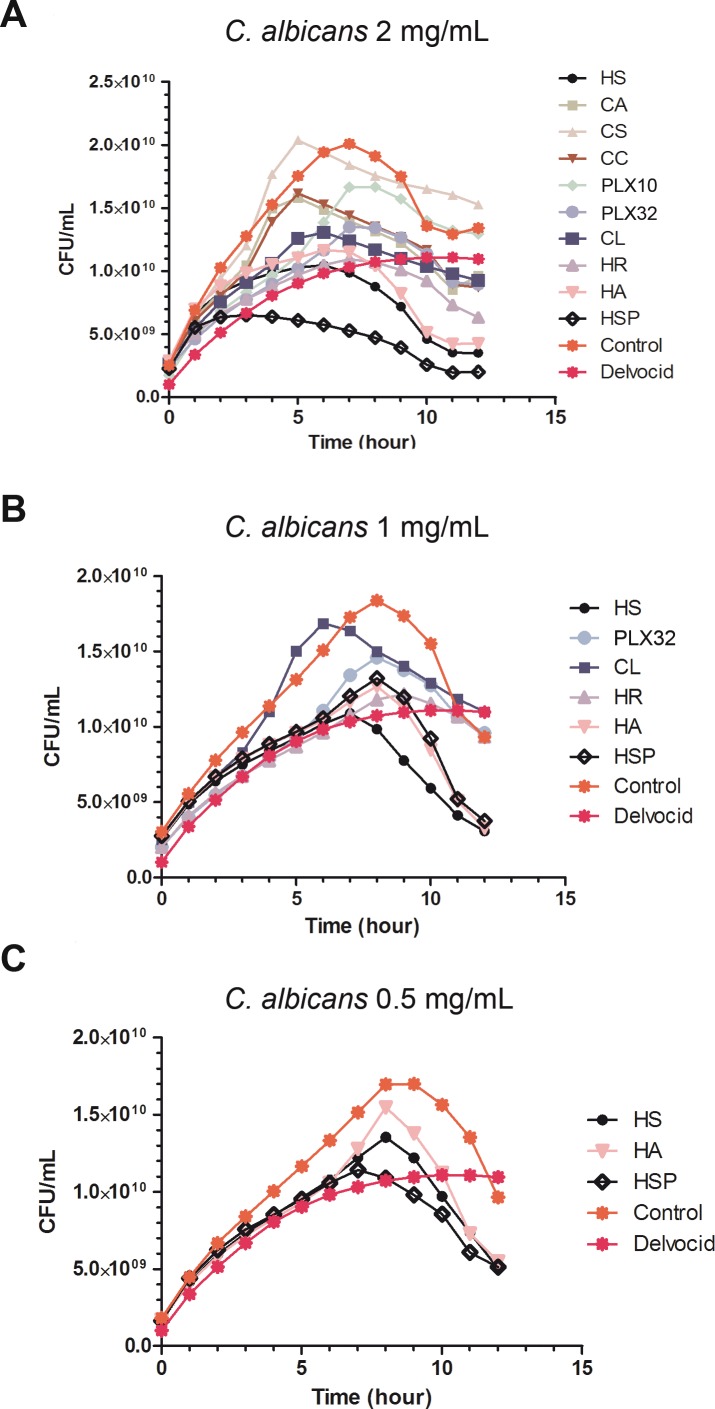
**Plots for MABA experiments against *C*. *albicans* yeast using different concentration of the extracts**: 2 mg/mL (A), 1 mg/mL (B) and 0.5 mg/mL (C). Points represent the mean of 12 replicates. Standard error was calculated for each point (data not shown).

Fig 4 shows the results for the inhibitory capacity of the extracts against *C*. *albicans*. The extracts HSP, HS, HR, HA, PLX32 and CL showed a significant inhibitory activity at 2 mg/ml compared to the control without inhibitory agent (Fig 4A). Some of the plant extracts, i.e. HS, HA and HSP revealed higher activity than the positive control with the antifungal DelvoCid®. When tested at a concentration of 1 mg/mL (Fig 4B), the extracts HA, HS and HSP still retained a good inhibitory activity against yeast growth. However, a significant temporal delay was detected when compared with first test at a concentration of 2 mg/mL. At 2 mg/mL, extracts started to reduce viability after 5 hours approximately, but this effect was observed after 7 hours when tested at 1 mg/mL (Fig 4B). HA, HS and HSP extracts were selected for testing at 0.5 mg/mL concentration (Fig 4C). Although all three extracts still exhibited strong activity on cell viability in the nutrient depletion phase, only HSP retained a similar activity than that shown by the positive control, i.e DelvoCid® a known yeast and molds inhibitor composed of natamycin.

### Characterization of the extracts by HPLC-DAD-ESI-IT-MS/MS analysis

**Fig 5:**
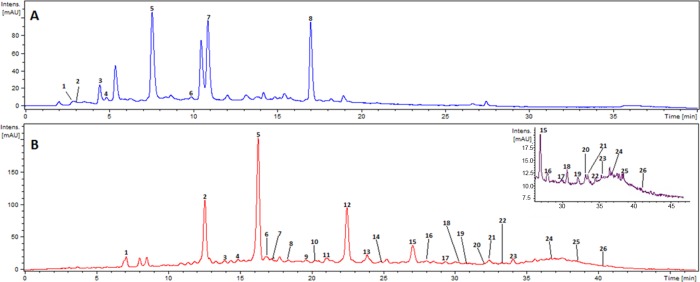
**HPLC chromatogram at 280 nm for HA (A) and CL (B) extracts.** Insert in B shows the amplified zone of the profile at 330 for more detailed peak identification. Numbers in the figure correspond to the peaks listed in Table 4 for HA and Table 5 for CL extracts respectively.

The polyphenolic composition of extracts HA and CL, which had not been previously characterized, was analyzed by HPLC-DAD-ESI-IT-MS/MS. HPLC profiles for these two extracts showing the main identified compounds are presented in Fig 5. A total of six compounds were tentatively identified by their mass spectra data in HA extract (Table 4), but two significantly abundant compounds could not be identified. The analysis of CL extract exhibited a total of 23 identified compounds and 3 more remained as unknown (Table 5). We have previously analyzed and reported the composition of the extracts derived from *Cistus* species (*C*. *ladanifer* and *C*. *salviifoliu*s) and *Hibiscus sabdariffa* in detail [11, 12, 20, 25]. The composition of *Lippia citriodora* and *Hypoxis rooperi* extracts has also been the subject of comprehensive studies reported by our group [14, 26, 27]. Information about the extracts is summarized in Table 2.

**Table 4:** MS data for HA extract.

Peak number	RT(min)	[M-H]^-^(m/z)	MS/MS (m/z)	Proposed compound	Reference
**1**	3	206.9	189, 127	Hydroxicitric acid	[28]
**2**	3.4	188.9	127	Hibiscus acid	[28]
**3**	4.5	386.8		Unknown	
**4**	4.8	363.3		Unknown	
**5**	7.5	352.9	285, 255	Chlorogenic acid	[20]
**6**	9.7	593.0	285	Kaempferol-3-O-rutinoside (isomer I)	[29]
**7**	10.9	352.9	285, 255	Chlorogenic acid	[20]
**8**	16.9	609.0	301, 179	Quercetin-3-rutinoside	[20]

Relevant chromatographic and MS data for the identification of the main peaks in HA extract.

**Table 5:** MS data for CL extract.

Peak number	RT (min)	[M-H]^-^(m/z)	MS/MS(m/z)	Proposed compound	Reference	
**1**	7.1	781	601,721	Punicalin		[13]
**2**	12.6	1081	780, 600	Punicalagin (isomer I)		[13]
**3**	14	509	461	Unknown		
**4**	14.8	525	489, 327	Unknown		
**5**	16.3	1081	780, 600	Punicalagin (isomer II)		[13]
**6**	16.9	371	209, 191 113	Hydroxy ferulic acid hexoside		[30]
**7**	17.4	445	401	Apigenin-7-O-glucuronide		[31]
**8**	18.2	431	223, 153	Apigenin-7-O-glucoside		[32]
**9**	20.5	525	489	Unknown		
**10**	21.1	221	-	Flavone		[33]
**11**	21.4	478	315, 363, 255	Isorhamnetin-3-O-galactoside		[34]
**12**	22.4	327	165,	Betulosidebetuloside		[11]
**13**	23.8	593	353	Kaempferol diglycoside		[13]
**14**	24.9	463	301,151	Quercetin glucoside		[34]
**15**	27.0	625	463, 301	Quercetin diglycoside		[30]
**16**	28.0	609	447, 285	Kaempferol diglucoside		[13]
**17**	29.3	565	316	Myricetin hexoside-malonate		[35]
**18**	30.2	533	285	Kaempferol hexoside-malonate (isomer I) (isomer II)		[35]
**19**	30.7	595	301	Quercetin diglycoside		[13]
**20**	32.0	463	301, 271, 255, 151	Quercetin-3-O-galactoside		[34]
**21**	32.4	505	-	Dimethyl ellagic acid glucuronide		[36]
**22**	33.2	493	319	Myricetin-3-O-glucuronide (isomer I)		[37]
**23**	34.1	493	319	Myricetin-3-O-glucuronide (isomer II)		[37]
**24**	36.9	339	177	Esculetin-O-glucoside		[34]
**25**	38.7	537	-	Ligstroside glucuronide		[38]
**26**	40.3	533	285	Kaempferol hexoside-malonate (isomer II)		[35]

Relevant chromatographic and MS data for the identification of the main compounds in CL extract.

### Correlation between HPLC analysis and antimicrobial activity

After the antimicrobial screening phase and the analysis of main polyphenolic compounds, all the data were analyzed in order to find a potential correlation between the presence of individual compounds and the activity against the tested microorganisms. For *S*. *aureus*, two (CS and CL) of the three most active extracts were enriched in ellagitannins and flavonols. Both families have demonstrated their antibacterial activity previously [9, 39]. The third one was HSP, which is a polyphenolic enriched extract of *H*. *sabdariffa* with a high polyphenolic content (28.42 ± 0.33 of gallic acid equivalents/100 g of dry weight) and with an important content in anthocyanidins as delphinidin and cyanindin sambubiosides and significant amounts of flavonols such as glycosylated quercetin and kaempferol derivatives. Both families, anthocyanidins [40, 41] and flavonols [8, 11, 42, 43], have demonstrated their activity against Gram positive bacteria previously. A recent study has proven the synergic antimicrobial effect between flavonols, ellagitannins and between flavonols and ellagitannins against *S*. *aureus* [13]. Therefore, these compounds may account for the stronger effect observed for CS, CL and HSP extracts. Anyhow, the presence of unknown or unidentified compounds could also contribute for such effect.

Ellagitannins and flavonols seem to be also related with *E*. *coli* antimicrobial activity, since CS and CL were also selected as the most active extracts against this microorganism. HSP was also very active, and exhibited the lowest endpoint value at 0.5 mg/mL (Fig 3C) so anthocyanidins and glycosylated flavonols also accounted for the antimicrobial activity against *E*. *coli* as occurred in *S*. *aureus*.

Finally, the antifungal activity against *C*. *albicans* seemed to be strongly related with the presence of either phenolic acids such as chlorogenic acid in the extracts (HA and HS) and the presence of anthocyanidins and flavonols (HS, HA and HSP).

## Discussion

Plants are undoubtedly one of the most promising sources for natural compounds with important biological activities. There are many examples of plant-derived molecules and plant extracts with activity against different microorganisms [6, 8, 43, 44]. Most of them are well chemically characterized but their mechanism of action remains unknown. However, in order to search for new antimicrobial drugs bearing wide spectrum antibacterial and antifungal activity, the development of fast and inexpensive methods that reduce time and costs for novel drug discovery is still required.

The main finding of the present work is the development of a fast, cheap and reliable method to screen antimicrobial activity of plant extracts against three different model microorganisms, both Gram positive and Gram negative, and yeast. This approach may be considered as a fast and inexpensive High Throughput Screening (HTS) method that can be applied in industries and laboratories. HTS methods are very demanded especially for pharmaceutical industries as they need fast methods to scrutinize hundreds of new molecules from synthetic or natural origin. The MABA method has been previously utilized for high-throughput screening of antimicrobial agents [18, 45] or for MIC50 determination in a variety of microorganisms as in [19, 23, 24], but suitability must be determined for each application and cell model [46]. Therefore, the present study provides additional information on the correlation between AlamarBlue® fluorescence results and CFU/mL data and validate this method for growth inhibitory tests of three different microorganisms such as *S*. *aureus*, *E*. *coli* and *C*. *albicans*, models for Gram positive, Gram negative and yeast respectively.

In the present study, ten plant extracts derived from different Mediterranean plants were analyzed for antimicrobial activities using MABA. The extracts belonged to very different plant families with different chemical composition (Table 2), as confirmed by HPLC-MS analysis. Some of these extracts have exhibited their antimicrobial activity previously, but they were never compared under the same conditions in order to select the most efficient ones against the studied microorganisms. The highest activity against *S*. *aureus* seemed to be related to the presence of ellagitannins and some specific flavonols, which were mostly present in *C*. *salviifolius* (ellagitannins and flavonols) and the polyphenol-enriched *H*. *sabdariffa* extract (flavonols). In contrast, *E*. *coli* inhibition was related more to the presence of other type of flavonols in *C*. *ladanifer*, but to ellagitannins and flavonols present in *C*. *salviifolius*. The polyphenol-enriched *H*. *sabdariffa* extract seemed to account for this activity. Concerning *C*. *albicans*, phenolic acids, anthocyanidins and flavonols present in the three extracts derived from *Hibiscus* genus are postulated to be the responsible compounds for such activity. The antifungal activity of chlorogenic acid has been previously reported [47–49], and there are some evidences about its mechanism of action, which seems to be related with the disruption of cell membrane structure and the subsequent loss of barrier function [48].

These results are in agreement to those previously reported which demonstrated the specificity of certain flavonols and ellagitannins either for Gram positive or for Gram negative bacteria [11, 13]. Plausibly big molecules such as ellagitannins may interact with bacterial membranes exerting their antimicrobial effects through a bacteriostatic mechanism [13], and/or inhibiting biofilm formation [50]. In contrast, monomeric flavonols may reach intracellular targets by passive diffusion through the cell-wall/membrane and exert more dramatic effects in bacterial metabolism.

Our findings also point out that the pharmacological interaction between the compounds in the extract may play an important role in the antimicrobial activity. In future studies it is important to consider not only the compounds present in a plant extract but also their exact proportions, since this may lead to synergistic interactions, as we have recently reported [13].

Our results demonstrate that the use of MABA in HTS methods is a promising tool when used in combination with potent analytic tools such as HPLC-DAD-ESI-MS/MS. Taking together, this strategy can be used in the future to screen and identify novel antibacterial and/or antifungal compounds from complex matrixes such as plant extracts. However, HPLC-MS analysis must be used in a second stage when the selection of the most active extracts has been achieved since this technique may become a bottleneck due to the difficulty to identify some compounds, as occurred here with HA and CL extracts.

In conclusion, the MABA described here can be successfully used to screen for antimicrobial activity of complex plant extracts. The results point out compound-specificity related to the antimicrobial activity against bacteria and fungi. Extracts enriched in ellagitannins and flavonols reveled promising antibacterial activity agents both against Gram positive and Gram negative bacteria. Phenolic acids, anthocyanidins and flavonols are plausibly more related to the antifungal activity.

Further research should be directed to discover the mechanism of action of the plant extracts studied here as well as to explore possible synergistic effect of these plant extracts with the existing antibiotics. In addition to their pharmacological use, plant extracts may have interesting applications in cosmetics and food industry. The increase of green chemistry and ecology has promoted the interest of consumers in products with no artificial additives. The use of natural compounds as an alternative to chemistry-based preservatives and antioxidants is currently in the portfolio of most cosmetics or food industries.

## Conclusions or Highlights

- AlamarBlue® can be used in HTS methods to screen for antimicrobial activity of botanical extracts.
- Extracts enriched in ellagitannins and flavonols are promising antibacterial agents both against Gram positive and Gram negative bacteria.
- Phenolic acids, anthocyanidins and flavonols may be related with the antifungal activity.
- The complementary antimicrobial activity between extracts derived from Hibiscus and Cistus genus deserves further research.
- The combination of MABA and HPLC-DAD-ESI-MS/MS allows to perform structure-activity correlations for natural antimicrobial compounds.

## Supporting Information

S1 Fig Correlation between the relative fluorescence units (RFU) and colony forming units (CFU). **A:** correlation between RFU and CFU for the Gram negative target-organism *E*. *coli WA321*, **B:** correlation between RFU and CFU for the Gram positive target organism *S*. *aureus 533R4*, **C:** correlation between RFU and CFU for the yeast-like fungi *C*. *albicans*.
(TIF)

S2 Fig **Additional controls for MABA in *S*. *aureus* (A), *E*.*coli* (B) and *C*. *albicans* (C).** The following antibacterial agents were applied in the positive assay controls: kanamycin (KM) 50 μg/mL, tetracycline (TC) 15 μg/mL, (Sigma-Aldrich product # K-4000, T-7660), ampicillin (AMPC) 50 μg/mL(GIBCO-Life Technologies cat# 11593–027). Delvocid® (DSM Food Specialties, The Netherlands) was used as antifungal agent and Cycloheximide and Thiobendazole in this case 200 mg/mL Cycloheximide or Delvocide were used, Thiobendazole was tested at a concentration of 25 mg/mL.
(TIF)
