# Supplementary figures and images for: Validation of the AlamarBlue® Assay as a Fast Screening Method to Determine the Antimicrobial Activity of Botanical Extracts
Source: PLoS One. 2016 Dec 29;11(12):e0169090. doi: 10.1371/journal.pone.0169090 (PMC5199036; doi:10.1371/journal.pone.0169090)

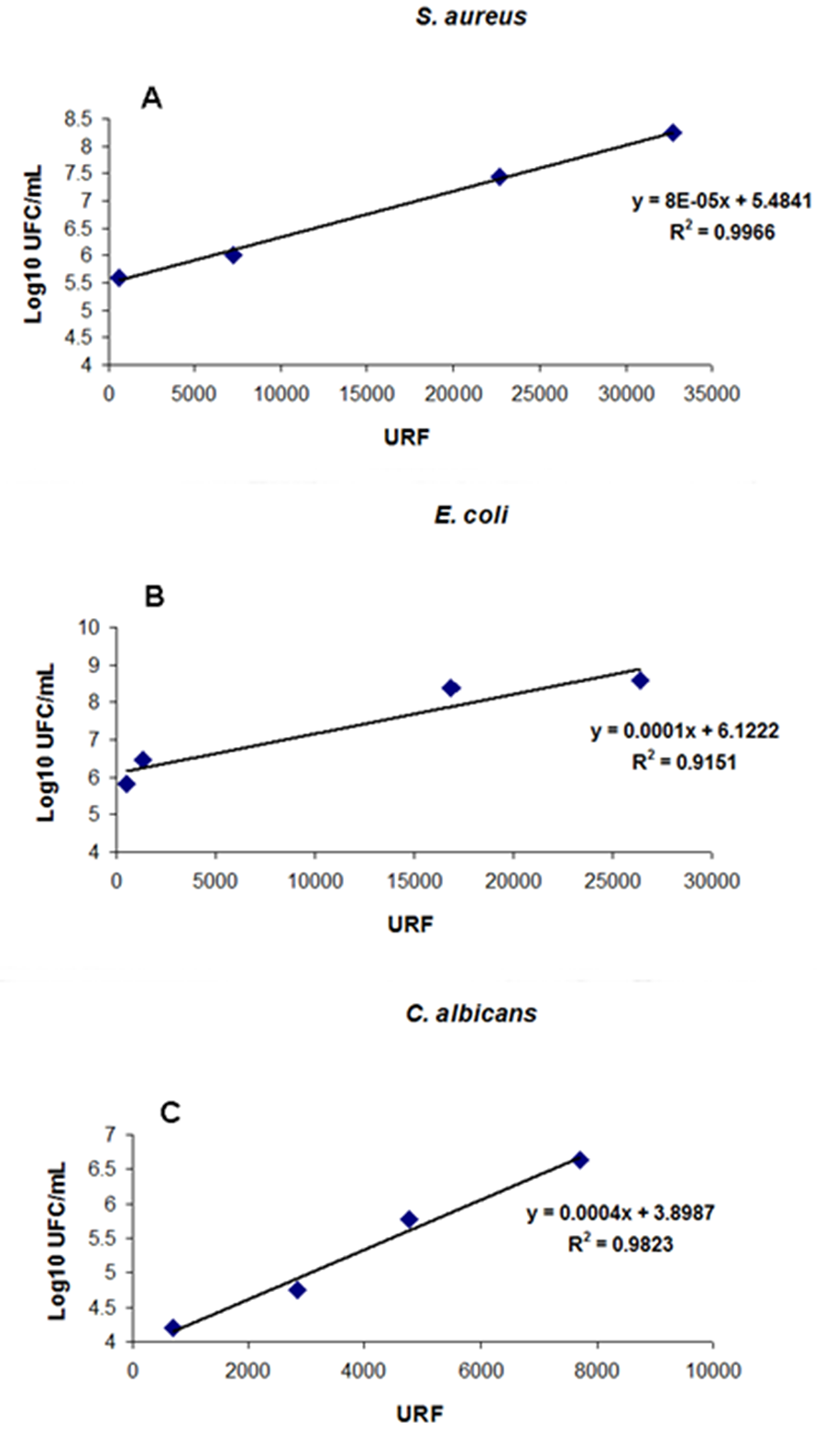

Supplement: S1 Fig — A: correlation between RFU and CFU for the Gram negative target-organism E. coli WA321, B: correlation between RFU and CFU for the Gram positive target organism S. aureus 533R4, C: correlation between RFU and CFU for the yeast-like fungi C. albicans. (TIF) [file pone.0169090.s001.tif]

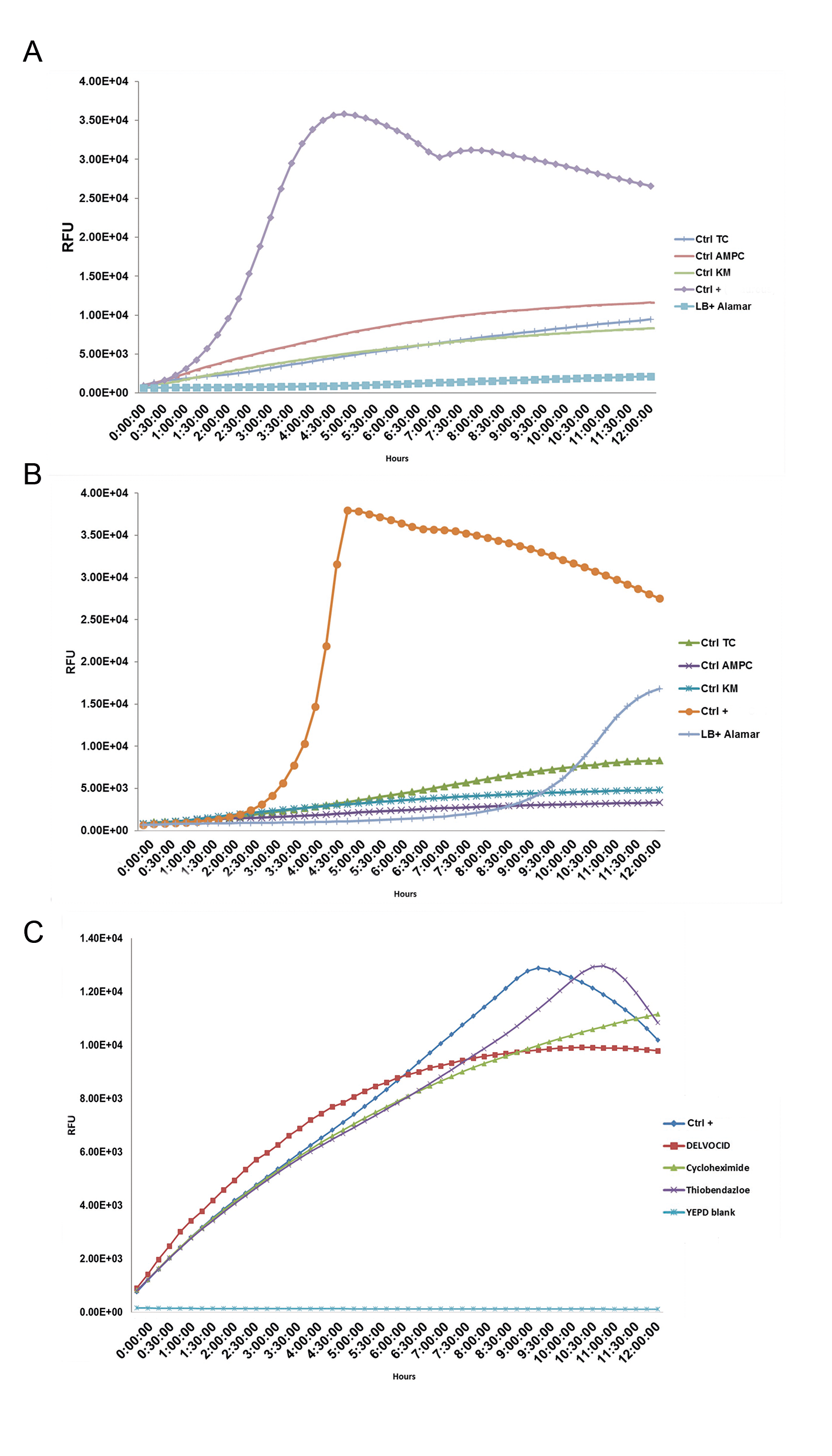

Supplement: S2 Fig — Additional controls for MABA in S. aureus (A), E.coli (B) and C. albicans (C). The following antibacterial agents were applied in the positive assay controls: kanamycin (KM) 50 μg/mL, tetracycline (TC) 15 μg/mL, (Sigma-Aldrich product # K-4000, T-7660), ampicillin (AMPC) 50 μg/mL(GIBCO-Life Technologies cat# 11593–027). Delvocid® (DSM Food Specialties, The Netherlands) was used as antifungal agent and Cycloheximide and Thiobendazole in this case 200 mg/mL Cycloheximide or Delvocide were used, Thiobendazole was tested at a concentration of 25 mg/mL. (TIF) [file pone.0169090.s002.tif]
